# Nanoparticle elasticity directs tumor uptake

**DOI:** 10.1038/s41467-017-02588-9

**Published:** 2018-01-09

**Authors:** Peng Guo, Daxing Liu, Kriti Subramanyam, Biran Wang, Jiang Yang, Jing Huang, Debra T. Auguste, Marsha A. Moses

**Affiliations:** 1 Vascular Biology Program, Boston Children’s Hospital, 300 Longwood Avenue, Boston, MA 02115 USA; 2 Department of Surgery, Harvard Medical School and Boston Children’s Hospital, 300 Longwood Avenue, Boston, MA 02115 USA; 3 Department of Biomedical Engineering, The City College of New York, 160 Convent Avenue, New York, NY 10031 USA; 4 School of Engineering and Applied Sciences, Harvard University, 29 Oxford Street, Cambridge, MA 02115 USA; 5 Present Address: Department of Chemical Engineering, Northeastern University, 360 Huntington Avenue, Boston, MA 02115 USA; 6 Present Address: Harvard-MIT Division of Health Sciences and Technology, Massachusetts Institute of Technology, 500 Main Street, Cambridge, MA 02139 USA; 7 Present Address: Department of Industrial & Systems Engineering, Texas A&M University, 101 Bizzell Street, College Station, TX 77843 USA

## Abstract

To date, the role of elasticity in drug delivery remains elusive due to the inability to measure microscale mechanics and alter rheology without affecting chemistry. Herein, we describe the in vitro cellular uptake and in vivo tumor uptake of nanolipogels (NLGs). NLGs are composed of identical lipid bilayers encapsulating an alginate core, with tunable elasticity. The elasticity of NLGs was evaluated by atomic force microscopy, which demonstrated that they exhibit Young’s moduli ranging from 45 ± 9 to 19,000 ± 5 kPa. Neoplastic and non-neoplastic cells exhibited significantly greater uptake of soft NLGs (Young’s modulus <1.6 MPa) relative to their elastic counterparts (Young’s modulus >13.8 MPa). In an orthotopic breast tumor model, soft NLGs accumulated significantly more in tumors, whereas elastic NLGs preferentially accumulated in the liver. Our findings demonstrate that particle elasticity directs tumor accumulation, suggesting that it may be a design parameter to enhance tumor delivery efficiency.

## Abstract (web summary)

Nanoparticle elasticity is thought to play an important role in drug delivery, but is little studied. Here, the authors use nanolipogels with tunable moduli to study the effect of particle elasticity on in vitro cellular uptake and in vivo tumor uptake, finding that stiffer particles are not as easily internalized.

## Introduction

Nanoliposomes (NLPs) and nanoparticles (NPs) are two major classes of drug delivery systems used to control drug distribution and release^1^. These two systems may be engineered with identical size, shape, and surface charge; however, there exists between them characteristic differences in their architecture and particle elasticity. NLPs have an aqueous core encapsulated within a lipid bilayer, whereas NPs are solid structures composed of amorphous and/or crystalline polymers or inorganic materials. Both NLPs and NPs exhibit size-dependent properties, significantly different from the properties of the bulk material.

Physical cues are known to govern cell–NP interactions^2,3^. One hundred nanometer particles more efficiently avoid the mononuclear phagocyte system (MPS), resulting in prolonged blood circulation^2,4,5^. Geometric shape regulates hemorheological dynamics, cellular uptake, and in vivo fate of NPs^6–8^. The particle aspect ratio exhibits up to ten times longer blood circulation than their spherical counterparts in rodents^7^. Surface charge also affects NP circulation lifetime and biodistribution^9^. Cationic particles showed significantly higher serum protein absorption and non-specific uptake by most cell lines compared to neutral or anionic particles^10^. Nonetheless, NPs engineered with optimal size, shape, and surface charge are limited to less than 1% tumor accumulation^11^.

Particle elasticity is hypothesized to alter cellular uptake and tumor accumulation due to its ability to bind cell surface receptors and squeeze through pores^12–19^. However, the ability to measure and tune NP elasticity while maintaining uniform size, shape, and surface chemistry has remained a challenge. Particle elasticity is often modulated by the extent of crosslinking however the surface properties (e.g., porosity and chemistry) also change. There is no mechanistic understanding of how particle elasticity governs cellular uptake. The results obtained from prior studies fail to address tumor accumulation. Today, particle elasticity is not yet a design parameter to enhance tumor delivery efficiency.

Herein, we synthesize a hybrid nanolipogel (NLG), an alginate encapsulating liposome, having a defined size, shape, and surface charge, with tunable elasticity. NLGs are a model system to investigate the effect of particle elasticity on both in vitro cellular uptake and in vivo tumor uptake. This study identifies the underlying mechanism that governs particle elasticity-mediated cellular uptake. We further provide the first experimental evidence regarding how particle elasticity regulates tumor uptake of NLGs in an orthotopic tumor model.

## Results

### Synthesis and characterization of NLGs with varying elasticity

**Fig. 1:**
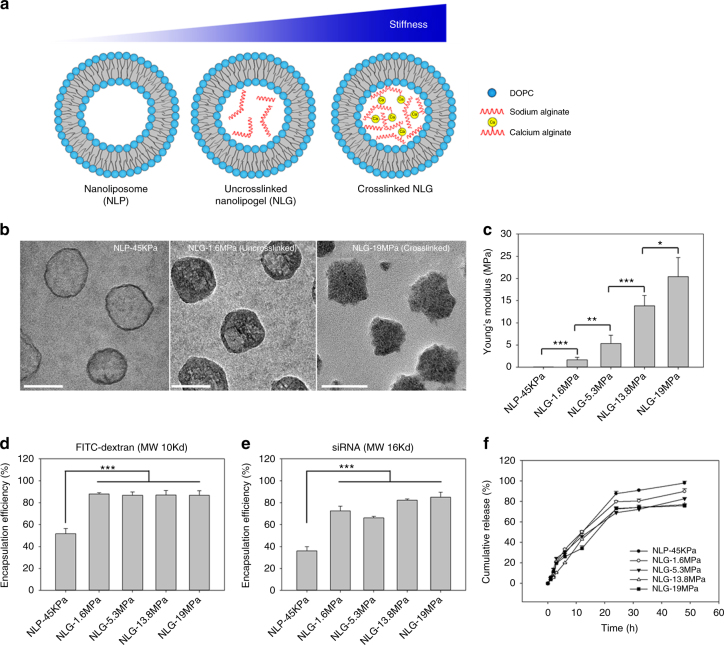
Synthesis and characterization of NLP and NLGs with varying elasticity. **a** Schematic illustration of nanoliposome–hydrogel complex system. NLP represents nanoliposome encapsulating PBS. Uncrosslinked NLG represents nanoliposome encapsulating uncrosslinked alginate (0 mM CaCl_2_). Crosslinked NLG represents nanolipogel encapsulating 1–5 mM CaCl_2_ crosslinked alginate. **b** The internal structure of NLP-45KPa, NLG-1.6MPa, and NLG-19MPa characterized by TEM. Scale bars represent 100 nm. **c** The Young’s moduli of synthesized NLP and NLGs characterized by AFM. The encapsulation efficiencies of FITC-dextran (**d**) and siRNA (**e**) in synthesized NLP and NLGs. **f** Sustained release profiles of synthesized FITC-dextran encapsulating NLP and NLGs. *P < 0.05, **P < 0.01, ***P < 0.001. The mean values and error bars are defined as mean and S.D., respectively

NLGs feature a core-shell structure with a lipid bilayer shell and a hydrogel core (Fig. 1a). The elasticity is modulated by the extent of crosslinking of the “core” material. Alginate undergoes coacervation, and its crosslinking degree is in a function of calcium concentration. The “shell”, or lipid bilayer, is consistent across all NLGs and controls the size of NLGs via extrusion method^20–22^. DOPC was chosen because it is zwitterionic and in the liquid phase at 37 °C. The rationale for using this model is to modulate particle elasticity independent of other physical cues (e.g., particle size, shape, and surface properties).

Size was characterized by dynamic light scattering (DLS) (Table 1). All NLP and NLGs exhibited similar hydrodynamic diameters of ~160 nm. The polydispersity indexes (PDIs) of these vehicles were less than 0.3, demonstrating uniformity. The zeta-potentials were slightly negatively charged. The interior structure was characterized by transmission electron microscopy (TEM). As seen in Fig. 1b, spherical NPs were formed. The presence of hollow lipid bilayers in NLPs (with an aqueous core) and a dense hydrogel core in NLGs are apparent.

**Table 1:** Dynamic light scattering characterization of synthesized NLP and NLGs with varying elasticity

Sample	Shell	Core	Crosslinker concentration (CaCl_2_, mM)	Size (nm)	Polydispersity index	Zeta potential (mV)
NLP-45KPa (w/o alginate)	DOPC	Saline	0	157 ± 55	0.247	−5.9 ± 3.6
NLG-1.6MPa	DOPC	Alginate	0	167 ± 40	0.205	−8.9 ± 2.2
NLG-5.3MPa	DOPC	Alginate	1	173 ± 42	0.272	−9.5 ± 1.2
NLG-13.8MPa	DOPC	Alginate	2.5	168 ± 38	0.164	−9.2 ± 1.1
NLG-19MPa	DOPC	Alginate	5	161 ± 39	0.226	−5.6 ± 2.5

The mean values and error bars are defined as mean and S.D., respectively

The Young’s modulus was determined by atomic force microscopy (AFM) (Fig. 1c). Particle elasticity correlates with calcium concentration. The NLPs without hydrogel core show the lowest Young’s modulus of 45 ± 9 kPa (NLP-45KPa). Uncrosslinked NLG (NLG-1.6MPa) and crosslinked NLGs (NLG-5.3MPa, NLG-13.8MPa, and NLG-19MPa) exhibited Young’s moduli ranging from 1.6 ± 0.6 MPa to 19 ± 5 MPa, ~40–400 folds higher than the elasticity of NLP, respectively. Altogether, NLG elasticity may be modulated independent of particle size, shape, and surface charge.

The encapsulation efficiency (EE) of FITC-dextran and scrambled siRNA were studied as two model drugs. As shown in Fig. 1d and e, all NLGs demonstrated significantly higher EEs of both FITC-Dextran and siRNA than NLP-45KPa. No significant change of EEs was measured among NLGs regardless of calcium concentration. This may be due to the polysaccharide network confining the Brownian diffusion of macromolecules causing retention within the lipid bilayer. The sustained release profiles demonstrated a slowed release of payload as a function of increasing particle modulus (Fig. 1f). Calcium crosslinking reduces the pore size of the hydrogel network and, in turn, affects release. These findings indicate that a polymeric core increases EE whereby the extent of crosslinking modulates release.

### Particle elasticity regulates in vitro cellular uptake

**Fig. 2:**
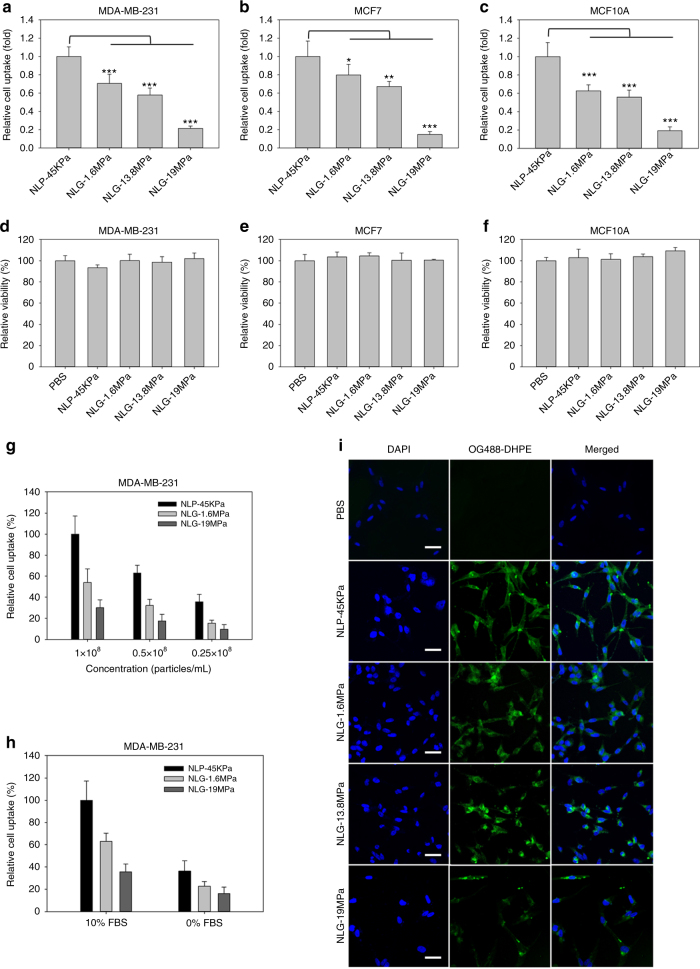
Particle elasticity regulates in vitro cellular uptake. Relative cellular uptake of synthesized NLP and NLGs by MDA-MB-231 (**a**), MCF7 (**b**), and MCF10A (**c**) cells. Relative cytotoxicity of engineered NLP and NLGs in MDA-MB-231 (**d**), MCF7 (**e**), and MCF10A (**f**) cells. The concentration (**g**) and serum (**h**) dependence of particle elasticity-mediated cellular uptake in MDA-MB-231 cells. **i** Fluorescent microscope images of MDA-MB-231 cellular uptake of synthesized NLP and NLGs with varying elasticity. The scale bars represent 50 μm. ***P < 0.001. The mean values and error bars are defined as mean and S.D., respectively

The cellular uptake of NLP-45KPa and NLGs by human breast cancer cells (MDA-MB-231 and MCF7) and normal human mammary epithelial cells (MCF10A) was studied. NLP-45KPa and NLGs, labeled with Oregon Green 488-1,2-Dihexadecanoyl-sn-Glycero-3-Phosphoethanolamine (OG488-DHPE, a fluorescent lipophilic dye, Ex/Em: ~496/524 nm), exhibited decreasing cellular uptake with increasing of the modulus, independent of cell type (Fig. 2a–c). The maximal cellular uptake occurred with NLP-45KPa (Young’s modulus, 45 ± 9 kPa), which is consistently 80% greater than NLG-19MPa. No cytotoxicity was observed across all samples (Fig. 2d–f). The cellular uptake and toxicity effects were consistent in murine breast cancer 4T1 cells (Supplementary Fig. 1).

We further evaluated the influence of particle concentration (Fig. 2g), serum (Fig. 2h), and incubation time (Supplementary Fig. 2) on particle elasticity-mediated cellular uptake. Although the baseline cellular uptake changed with varying test conditions, the elasticity dependent trend for uptake was independent of particle concentration, serum or incubation time.

Fluorescent microscope images (Fig. 2i) confirmed that particle elasticity modulates cellular uptake. As seen in the fluorescent images of MDA-MB-231 cells treated with NLP-45KPa, internalization was high and uniformly dispersed in the cytoplasm. In contrast, when treated with NLG-19MPa, significantly fewer NLG-19MPa particles were internalized and localized in the perinuclear region, indicating that they were trapped in endosomes or lysosomes as a result of endocytosis. This particle internalization pattern shift with increasing particle elasticity was also observed in MCF7 cells (Supplementary Fig. 3).

**Fig. 3:**
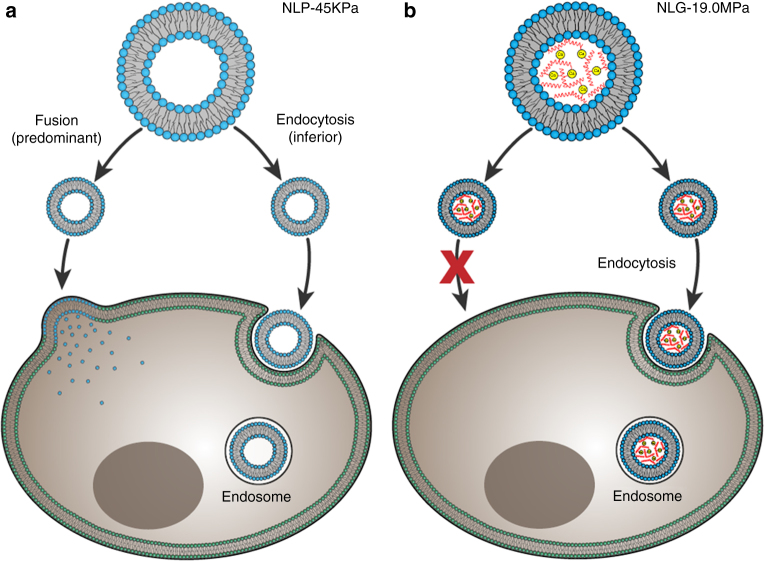
Cell internalization pathway shift by varying particle elasticity. Soft NLP-45KPa (**a**) enters the cell via two pathways: fusion (predominant) and endocytosis (inferior). Hard NLG-19MPa (**b**) enters cell via only clathrin-mediated endocytosis

There are two possible explanations for this cell internalization pattern shift. First, NLPs could enter the cell via different pathways (e.g., fusion) other than endocytosis. Second, endocytosed NLP could exhibit faster release within the cytoplasm than elastic NLGs. Previous studies have revealed that the lipid bilayer of NLPs can readily fuse with the plasma membrane of cells; this fusion process requires less time and energy than endocytosis^23–25^. We therefore hypothesized that particle elasticity may mediate cellular uptake by regulating cell internalization pathways. In our hypothesis, as illustrated in Fig. 3, soft NLP-45KPa may enter the cell through two independent pathways: fusion and endocytosis. Fusion is more dominant than endocytosis due to its low-energy requirement^26^. In comparison, the elastic NLGs may enter the cell only through endocytosis, which necessitates membrane bending, surface tension, and coated pits; thus, cells take more time and energy to internalize the same amount of NLG-19MPa as opposed to NLP-45KPa.

**Fig. 4:**
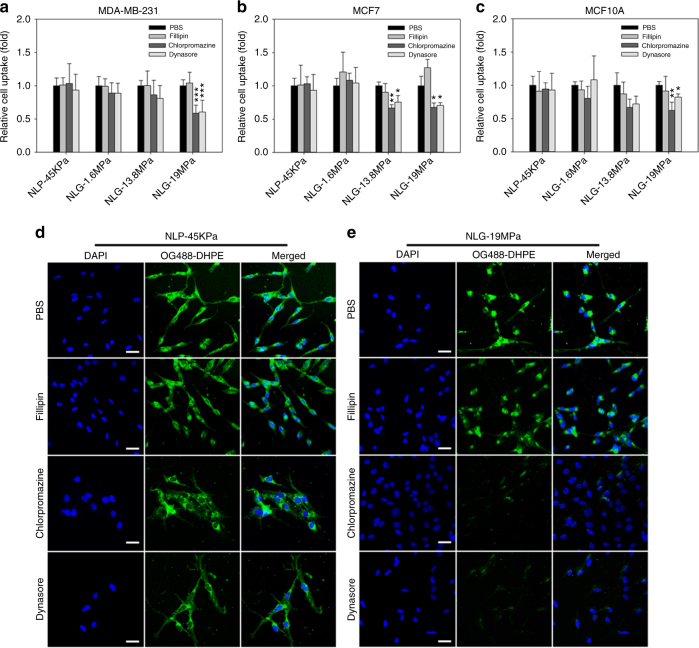
Particle elasticity mediates cellular uptake via different internalization pathways. Relative cellular uptake of NLP and NLGs in MDA-MB-231 (**a**), MCF7 (**b**), and MCF10A (**c**) cells in the presence of small molecules inhibiting clathrin (Chlorpromazine), caveolae (Filipin) and both endocytic pathways (Dynasore). Representative fluorescent microscope images of cellular uptake of soft NLP-45KPa (**d**) and hard NLG-19MPa (**e**) in response to endocytosis inhibitors. The scale bars represent 50 μm. *P < 0.05, **P < 0.01, ***P < 0.001. The mean values and error bars are defined as mean and S.D., respectively

To validate this hypothesis, three endocytosis inhibitors (Filipin, Chlorpromazine, and Dynasore) were used to inhibit caveolae-mediated, clathrin-mediated, and caveolae/clathrin-mediated endocytosis, respectively. Cellular uptake of NLP-45KPa was not affected by any of the endocytosis inhibitors (Fig. 4a–c). This is due to NLP-45KPa entering cells predominantly via fusion, which is not affected by endocytosis inhibition (Fig. 4d). In contrast, the internalization of NLG-19MPa was significantly inhibited by Chlorpromazine and Dynasore but not Filipin (Fig. 4e). These results indicate that the internalization of NLG-19MPa strictly depends on clathrin-mediated endocytosis. This is also consistent in murine neoplastic 4T1 cells (Supplementary Fig. 4). Particle elasticity modulates cellular uptake via distinct cell internalization pathways.

### Particle elasticity regulates in vivo tumor uptake

**Fig. 5:**
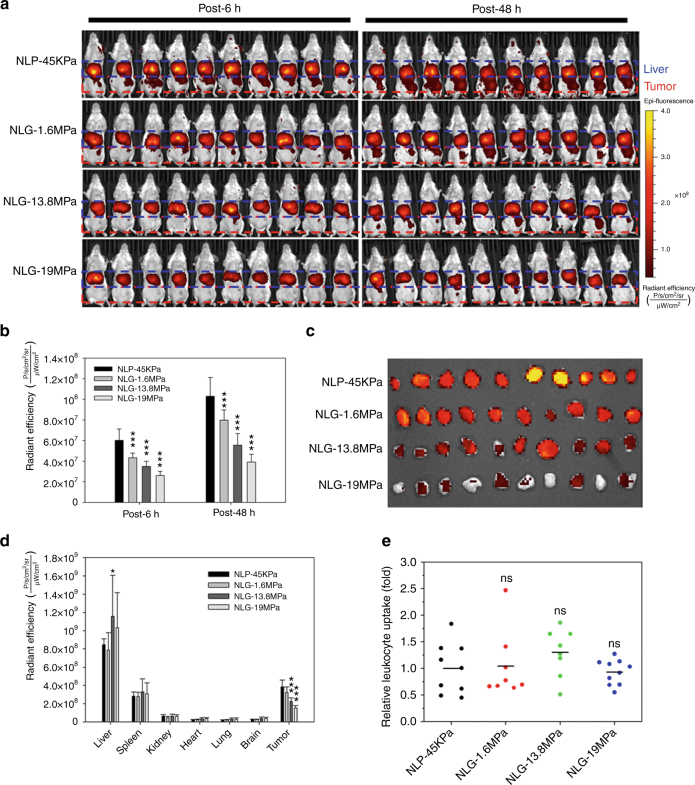
Particle elasticity regulates in vivo tumor uptake. **a** In vivo NIR fluorescent images of mice at post-6h and -48h after i.v. administration of DiR-labeled NLP-45KPa, NLG-1.6MPa, NLG-13.8MPa, and NLG-19MPa (*n* = 10 for each group). **b** In vivo tumor accumulation of synthesized NLP-45KPa and NLGs was quantified by fluorescent intensity. **c** Ex vivo NIR fluorescent images of excised tumors at post 48 h. **d** The biodistribution of NLP-45KPa and NLGs in organs (liver, spleen, lung, kidney, heart, and brain) and tumors was quantified by their fluorescent intensity at post-48h (*n* = 10 for each group). **e** Uptake of NLP-45KPa and NLGs by mouse leukocytes was quantified by flow cytometry (*n* = 8–10 for each group). ns not significant, *P < 0.05, **P < 0.01, ***P < 0.001. The mean values and error bars are defined as mean and S.D., respectively

To determine whether particle elasticity impacts tumor uptake and biodistribution in vivo, near infrared (NIR) lipid dye, lipophilic carbocyanine DiIC18 (DiR)-labeled NLP-45KPa and NLG particles were injected via the tail vein in an orthotopic, syngeneic, 4T1 breast tumor model. Tumor uptake was measured via NIR fluorescent imaging at 6 and 48 h post injection; the tumor accumulation of NLP-45KPa and NLGs inversely correlated with increasing particle elasticity, consistent with the in vitro cellular uptake data. At 48 h, NLP-45KPa demonstrated the highest tumor accumulation (Fig. 5a and b), exhibiting a 1.3, 1.9, and 2.6-fold increase relative to NLG-1.6MPa, NLG-13.8MPa, and NLG-19MPa, respectively. Particles with higher moduli were less likely to accumulate into tumors.

The in vivo tumor uptake data obtained from live mice precisely matched ex vivo NIR fluorescent images of excised tumors (Fig. 5c). Figure 5d shows the particle accumulation in six organs (liver, spleen, lung, kidney, brain, and heart) and 4T1 tumors harvested from mice at 48 h after a single tail vein administration. Of the six organs analyzed, the liver and spleen were the major accumulation sites of all particles. Notably, the liver uptake of NLGs with Young’s moduli above 13.8 MPa is relatively higher than that of NLP-45KPa and NLG-1.6MPa (Fig. 5d; Supplementary Fig. 5). Tumor uptake was significantly increased in NLP-45KPa and NLG-1.6MPa, suggesting that decreasing elastic modulus preferentially enhances both cellular internalization and permeation of tumors.

We further investigated the mouse immune system interaction with NLP-45KPa and NLGs as a function of modulus by measuring mouse leukocyte uptake of NLP-45KPa and NLGs. Mouse blood was collected at 48 h post injection of DiR-labeled NLP-45KPa or NLGs, and leukocytes were isolated from the blood by red blood cell lysis and centrifugation. Leukocyte uptake was quantified using flow cytometry (Fig. 5e); no significant difference between different treatment groups was observed. This is most likely due to particles being internalized by leukocytes via non-specific phagocytosis^27^. To our knowledge, the in vivo data presented here is the first experimental evidence of particle moduli regulating in vivo particle uptake within orthotopic tumors.

## Discussion

We synthesized a hybrid NLP-hydrogel vehicle that allows one to alter particle modulus independently of particle size, shape, and surface chemistry. The particle moduli varied from 45 ± 9 kPa to 19 ± 5 MPa, which corresponded to up to a fivefold in vitro cellular uptake and 2.6-fold in vivo tumor uptake. The underlying mechanism of the modulus-mediating cellular response is due to an internalization pathway shift from fusion (low-energy dependence) to endocytosis (high-energy dependence). This cell internalization pathway shift can be switched by altering particle elasticity.

Other mechanisms may also contribute to the particle elasticity-mediated tumor uptake, such as the extravasation capability^28,29^. We therefore investigated the extravasation capability of synthesized NLGs with tunable elasticity via a nanoporous membrane deformability assay^29^ (Supplementary Fig. 6) and an endothelial barrier assay^28^ (Supplementary Fig. 7). The difference in extravasation between NLP-45KPa and NLGs is moderate (~15%), not as dramatic as their cellular uptake differences (~80%). We postulate that particle elasticity-mediated cell internalization pathway is the dominant mechanism for regulating in vivo tumor uptake. It is noteworthy that Jiang and Shi et al. have systematically investigated the elasticity-mediated cell and tumor uptake of lipid-coated poly(lactic-co-glycolic) acid (PLGA) NPs, and they reported that rigid lipid-coated PLGA NPs enter cell more efficiently than soft ones^18,19^. This is potentially due to the fact that their lipid-coated PLGA NPs are significantly more rigid (Young’s modulus range: 0.76–1.20 GPa) than the NLP-45KPa and NLGs tested in our studies (Young’s modulus range: 0.045–19 MPa), and at such high elasticity, clathrin-mediated endocytosis becomes the dominant cell internalization pathway for both rigid and soft lipid-coated PLGA NPs, while fusion pathway is negligible for these particles^18^. It suggests that the important role of fusion pathway in particle elasticity-mediated cell internalization is limited to a low particle elasticity (<19 MPa), and cannot be applied to highly elastic NPs (higher than 0.76 GPa).

From a broader perspective, our findings that particle modulus regulates cell internalization pathways may have other important implications. Most recently, the CRISPR–Cas9 system has emerged as a revolutionary gene editing tool with tremendous potential in genetic disease therapy^30–32^. However, like other gene-based therapeutics (e.g., siRNA and plasmid), the utility of CRISPR–Cas9 is limited by its rapid biodegradation and inefficient systemic delivery^33–35^. From a practical point of view, our findings that NLPs fuse with the cell membrane and avoid endosomes create an opportunity for intracellular delivery.

NLPs may mimic nature’s method for drug delivery. Extracellular vesicles (e.g., exosomes), a naturally occurring drug delivery system, with similar size and structure to our engineered soft NLPs, provide efficient delivery of proteins and RNAs to facilitate intercellular communication in a range of diverse and yet unknown cellular processes^36–38^. We can deduce that extracellular vesicles may take advantage of low particle moduli to protect their contents from endosome/lysosome degradation^39,40^.

In conclusion, particle modulus directly regulates cellular uptake of drug delivery vehicles via regulating cell internalization pathways. Our in vivo results also provide strong evidence that particle elasticity governs tumor uptake of systemically administered NPs. Taken together, our findings provide critical insight into the role of biomechanical cues between particles and cells and between particles and organs. This work may assist in the design of more efficient particles for therapeutic and diagnostic applications.

## Methods

### Cell culture

Human breast cancer MDA-MB-231 and MCF7 cells, human non-neoplastic mammary epithelial MCF10A cells, murine breast cancer 4T1 cells, and human microvascular endothelial cells (HMVECs) were purchased from American Type Culture Collection (ATCC, Manassas, VA, USA). MDA-MB-231 and MCF7 cells were cultured in DMEM, 4T1 cells in RPMI-1640 Medium, MCF10A cells in DMEM/F12 (1:1), and HMVECs in Lonza EGM-2-MV with recommended supplements, respectively. All cells were cultured in a 37 °C humidified incubator with 5% CO_2_.

### Preparation of NLP and NLGs

NLP and NLGs were prepared using extrusion method with modifications^20–22^. Overall, 50 μmol 1,2-dioleoyl-sn-glycero-3-phosphocholine (DOPC) was dried under a stream of nitrogen gas and stored in a vacuum desiccator overnight, the resulting phospholipid film was resuspended in 1 mL DMSO:EtOH (7:3, v:v). This solution was added dropwise through a syringe (gauge size 20) to a solution of 9 mL PBS or 1 mg/mL sodium alginate aqueous solution under vigorous stirring. The resulting lipogel NP solution went through five freeze-thaw cycles and was repetitively extruded through a 200 nm polycarbonate track etch nanoporous membrane for five times. The resulting NLP or NLG solution was dialyzed in FLOAT-A-LYZER G2 dialysis tubing (MWCO 1,000 kDa) in deionized H_2_O overnight at 4 °C. After dialysis, an ionic crosslinking solution containing (0–10 mM CaCl_2_) was added dropwise to NLG solution (1:1, v:v) in order to crosslink the sodium alginate monomers within the liposomal membrane. After 1 h incubation, the resulting NLG solution was further dialyzed in FLOAT-A-LYZER G2 dialysis tubing (MWCO 1000 kDa) in PBS for 24 h at 4 °C. In drug encapsulation experiments, FITC-dextran and siRNA-encapsulating NLP and NLGs were prepared using the similar procedure except adding 1 mg/mL FITC-dextran or 15 μg/mL siRNA to the 9 mL PBS or alginate solution. In the cellular and tumor uptake experiments, OG488-DHPE or DiR-labeled NLP and NLGs were prepared using the similar procedure except adding 0.2 mol% OG488-DHPE or 1 mol% DiR to 1 mL lipid solution.

### In vitro cytotoxicity assay

The in vitro cytotoxicity of NLP and NLGs with different elasticity was evaluated with human neoplastic and non-neoplastic cells^41^. Briefly, 10^4^ cells (MDA-MB-231, MCF7, or MCF10A) were seeded in each well of a 96-well plate and incubated for 24 h. Cells were treated with NLP or NLGs with different elasticity at 10^8^ particles/mL in DMEM with 10% FBS for another 24 h. Cells were rinsed twice with PBS and further grown for 48 h. The cell viability was determined by a Dojindo cell viability assay using the protocol from the manufacturer (Rockville, MD, USA).

### In vitro cellular uptake measurement

Overall, 10,000 cells (MDA-MB-231, MCF7, or MCF10A) were seeded in each well of a 96-well plate and allowed to attach overnight. To determine the cellular uptake of NLP and NLGs with different elasticity, the cells were incubated with OG488 DHPE-labeled NLPs or NLGs at a concentration of 10^8^ particles/mL in DMEM with 10% FBS for 4 h. Cells were then washed with PBS twice and the fluorescence of each well on the 96-well plate was quantified by SpectraMaxGEMIN XPS fluorescence spectrophotometer (Molecular Devices Corp, Sunnyvale, CA, USA) at 501/526 nm excitation/emission.

### Orthotopic tumor model

Animal experiments were performed according to the protocols approved by the Institutional Animal Care and Use Committees of City College of New York. A total of 1 × 10^6^ mouse breast cancer 4T1 cells were orthotopically injected into the fourth mammary fat pad of female BALB/c mice (*n* = 40, Charles River, Wilmington, MA, USA)^42^. For in vivo NIR fluorescent imaging experiments, tumors grew for 2–3 weeks until they were at least 200 mm^3^ in volume. Mice were randomized into four treatment groups (*n* = 10 for each group), which were injected i.v. with (1) DiR-NLP-45KPa, (2) DiR-NLG-1.6MPa, (3) DiR-NLG-13.8MPa, (4) DiR-NLG-19MPa (at dosage of 20 mg lipids/kg mouse weight). At 6 h and 48 h after the injection, in vivo fluorescence imaging was performed on tumor-bearing mice using an IVIS Lumina II system (Caliper, Hopkinton, MA, USA). At 48 h post injection, the mice were sacrificed via cervical dislocation and blood was collected immediately via cardiac puncture. The NIR fluorescence intensity of various excised organs (brain, heart, liver, lung, kidney, spleen, and tumor) was measured by IVIS Lumina II system. The mouse leukocytes were isolated from mouse whole blood via centrifugation at 1200 rpm for 15 min, and followed by removing mouse red blood cells using RBC lysis buffer. The fluorescence intensity of leukocytes treated with different DiR-labeled NLP and NLGs were quantified using a BD FACSCalibur Flow Cytometer (BD Biosciences, San Jose, CA, USA).

### Statistical analysis

All of the experimental data were obtained in triplicate unless otherwise mentioned and are presented as mean ± S.D. Statistical comparison by analysis of variance was performed at a significance level of *P* < 0.05 based on a Student’s *t*-test.

### Data availability

The data that support the findings reported herein are available on reasonable request from the corresponding authors.

## Electronic supplementary material

Supplementary Information
