# Supplementary material for: Nanoparticle elasticity directs tumor uptake
Source: Nat Commun. 2018 Jan 9;9:130. doi: 10.1038/s41467-017-02588-9 (PMC5760638; doi:10.1038/s41467-017-02588-9)
Supplement: Supplementary file 1 — Supplementary Information [file 41467_2017_2588_MOESM1_ESM.pdf]

Supplementary Methods

Materials

1,2-dioleoyl-sn-glycero-3-phosphocholine (DOPC) was purchased from Avanti Polar Lipids (Alabaster, AL, USA). Sodium alginate was purchased from FMC BioPolymer (Philadelphia, PA, USA). Whatman Nuclepore track-etched membranes, FITC-dextran, Attachment Factor Solution, Chlorpromazine, Filipin, and Dynasore were purchased from Sigma Aldrich (St. Louis, MO, USA). FLOAT-A-LYZER G2 dialysis tubing (MWCO 1,000 KD) was purchased Spectrum Labs (Rancho Dominguez, CA, USA). 300-mesh carbon-coated copper grid was purchased from Ted Pella (Redding, CA, USA). Oregon Green 488 1,2-Dihexadecanoyl-sn-Glycero-3-Phosphoethanolamine (OG488-DHPE), Quant-iT RiboGreen RNA Assay Kit, 4',6-Diamidino-2-Phenylindole, Dihydrochloride (DAPI), Transwell Permeable Supports, Lab-Tek II Chamber Slide System, Lonza EGM-2-MV, and lipophilic carbocyanine DiOC18 (7) (DiR) were purchased from Thermo Fisher Scientific (Waltham, MA, USA). Control siRNA was purchased from GE Dharmacon (Lafayette, CO, USA). Gibco® Dulbecco's Modified Eagle Medium (DMEM), Gibco®DMEM/F12(1:1) were purchased from Invitrogen (Carlsbad, CA, USA). Fluorogel with tris buffer was purchased from Electron Microscopy Sciences (Hatfield, PA, USA). Red blood cell (RBC) Lysis Buffer was purchased from Biolegend (San Diego, CA, USA). Dojindo Cell Counting Kit-8 was purchased from Dojindo Molecular Technologies (Rockville, MD, USA). Fetal bovine serum (FBS) purchased from Atlanta Biologicals (Flowery Branch, GA, USA).

Atomic force microscopy (AFM)

The elasticity of synthesized NLP-45KPa and NLGs was quantified by AFM force measurement using a previously reported method with modifications[1,2]. The elasticity of all the samples was measured in solution (water).

Sample patch preparation: Freshly cleaved mica disc (15 mm in diameter, Electron Microscopy Sciences, Hatfield, PA, USA) was mounted onto the bottom of a petri dish using a sticky tab, and fresh prepared NLP-45KPa or NLGs solution in PBS (5 µL) was incubated on the mica surface for 1 hr at RT for sample absorbance. After this, unbound sample solution on mica disc was removed by a pipette, and the Mica disc was immersed in water and measured immediately.

AFM measurements: AFM force measurements were performed on an Asylum MFP-3D SA AFM (Asylum Research, Santa Barbara, CA, USA), with silicon nitride, four sided pyramid tips (BL-TR400PB-35, Asylum Research). The spring constant of the tips was calibrated every time by the Thermal Method, and all tips used have a spring constant between 0.02 and 0.04 N/m. The AFM was performed in contact mode, with a trigger voltage of 0.5V. The Young's modulus was determined by fitting the force-distance curve by the Hertz equation. The quasi-static force F is related to the indentation depth δ:

$$F = \frac{E}{1-\nu^2}\frac{\tan\beta}{\sqrt{2}}\delta^2 \quad (1)$$

Where β is the face angle, ν is Poisson's ratio of the material assumed here to be 0.5 (isotropic incompressible), and E is the Young's modulus. For all the samples, at least 5 force curves are used to get statistic value of elastic properties.

Characterization of NLP and NLGs

The size and surface charge of NLGs with varying elasticity were determined by dynamic light scattering measurement on a Zeta-PALS analyzer (Brookhaven Instruments, Holtsville, NY, USA) in PBS (pH 7.4). The morphology of NLP and NLGs was determined by transmission electron microscope (TEM) imaging on a JEOL 2100 TEM (JEOL USA Inc, Peabody, MA, USA). Freshly prepared NLP and NLGs nanoparticles (5 μL) were carefully dropped onto a 300-mesh carbon-coated copper grid (Ted Pella, Inc., Redding, CA, USA) and allowed to stand at room temperature for 5 min. All images were acquired at an accelerating voltage of 100 kV.

Encapsulation efficiency measurement

After preparing FITC-dextran or control siRNA encapsulating NLP or NLGs with different elasticity, the unencapsulated FITIC-dextran or siRNA were removed by dialysis using FLOAT-A-LYZER G2 dialysis tubing (MWCO 300 KDa) in PBS for 24 hr at 4 °C. Next, 20 μL dialyzed NLP or NLG samples was added to 1 mL of 0.5% Triton X-100 in a microcentrifuge tube and vortexed for 1 min to disrupt the structure of lipid bilayer. This microcentrifuge tube was incubated at 37°C for 1 hr. The released FITC-dextran was directly measured using a Synergy H4 Microplate reader (Winooski, VT, USA) at 490/525 nm excitation/emission with its standard concentration curve. The release siRNA was measured by the Quant-iT RiboGreen RNA reagent

according to the manufacturer's instructions at 485/520 nm excitation/emission. Each sample was assayed in triplicated. The encapsulation efficiency (%) is the encapsulated FITC-dextran or siRNA concentration divided by the initial FITC-dextran or siRNA concentration and multiplied by 100.

Sustained release profile measurement

Release of FITC-Dextran from NLP and NLGs with varying elasticity was carried out in PBS at pH 7.4. The FITC-Dextran encapsulating NLP or NLG solution (1 mL, 200 μg/mL) was added to a FLOAT-A-LYZER G2 dialysis tubing (MWCO 300 KDa). The dialysis tube was placed in a beaker with 50 mL PBS (pH 7.4). Then the beaker was sealed with parafilm and incubated at 37°C on a shaker (100 rpm). For each time point, three 100 μL samples were collected from the solution outside of dialysis tube and the fluorescence intensity was measured on a SpectraMaxGEMIN XPS fluorescence spectrophotometer (Molecular Devices Corp, Sunnyvale, CA, USA). The release rate of FITC-Dextran was calculated based on a standard fluorescence concentration calibration curve.

Fluorescent cell imaging

100,000 cells (MDA-MB-231, MCF7, or MCF10A) were seeded in 8-well Lab-Tek II Chamber Slide System and allowed to attach overnight[3,4]. Cells were incubated with OG488-DHPE labeled NLPs or NLGs at a concentration of 10^8 particles/mL in DMEM with 10% FBS for 4 hrs at 37°C. DAPI was used to stain the cell nucleus. After NLP or NLG treatment, cells were fixed by 4% Formalin solution and dried overnight in the dark, and fluorescent images of stained cells were obtained using a Leica TCS SP5 confocal fluorescent microscope (Buffalo Grove, IL, USA).

Endocytosis inhibitor treatment

Three endocytosis inhibitors (Chlorpromazine, Filipin, and Dynasore) were used in our study to investigate the cellular uptake pathways of NLP and NLGs with different elasticity. Briefly, MDA-MB-231, MCF7, or MCF10A cells were seeded overnight and pre-incubated with these endocytosis inhibitors separately: chlorpromazine at 10 μM (clathrin mediated endocytosis), fillipin at 5 μM (caveolae mediated endocytosis) and dynasore 100 at μM (clathrin and caveolae mediated endocytosis) for 30 min at 37°C before NLP or NLG treatments.

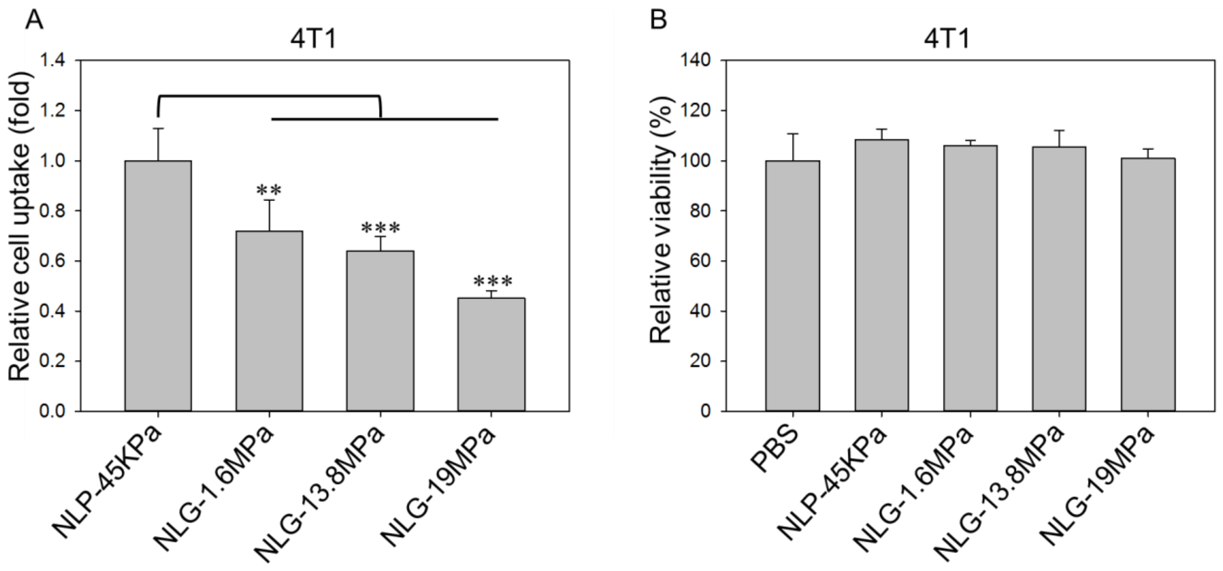

Supplementary Figure 1. The cellular uptake (A) and cytotoxicity (B) of synthesized NLP and NLGs in murine neoplastic 4T1 cells. ** P<0.01, *** P<0.001. The mean values and error bars are defined as mean and S.D., respectively

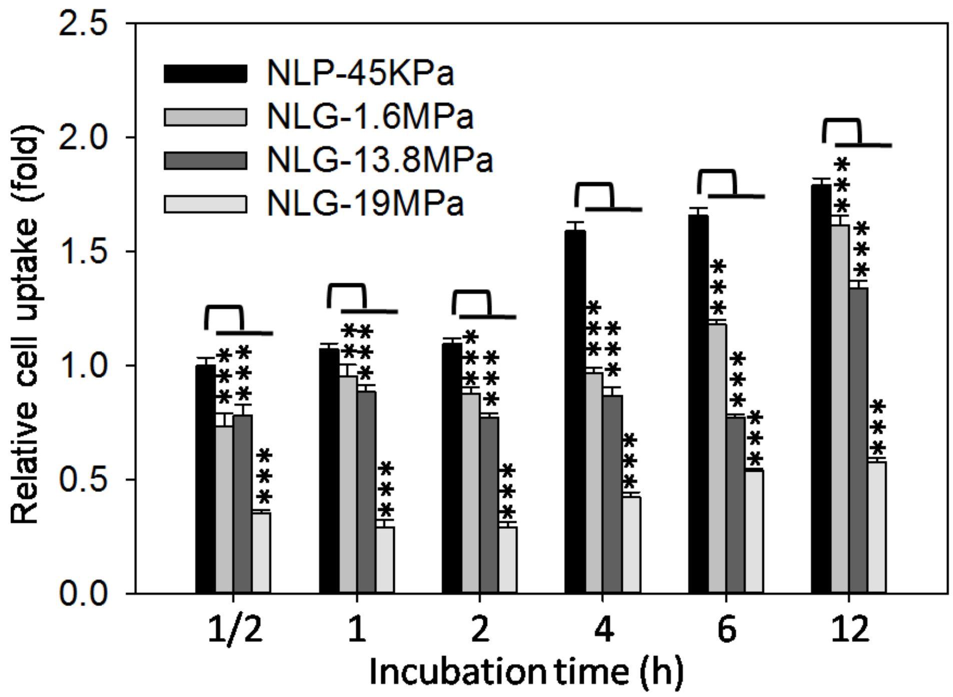

Supplementary Figure 2. MDA-MB-231 cellular uptake of NLP-45KPa, NLG-1.6MPa, NLG-13.8MPa, and NLG-19MPa at different time points. ** *P*<0.01, *** *P*<0.001. The mean values and error bars are defined as mean and S.D., respectively

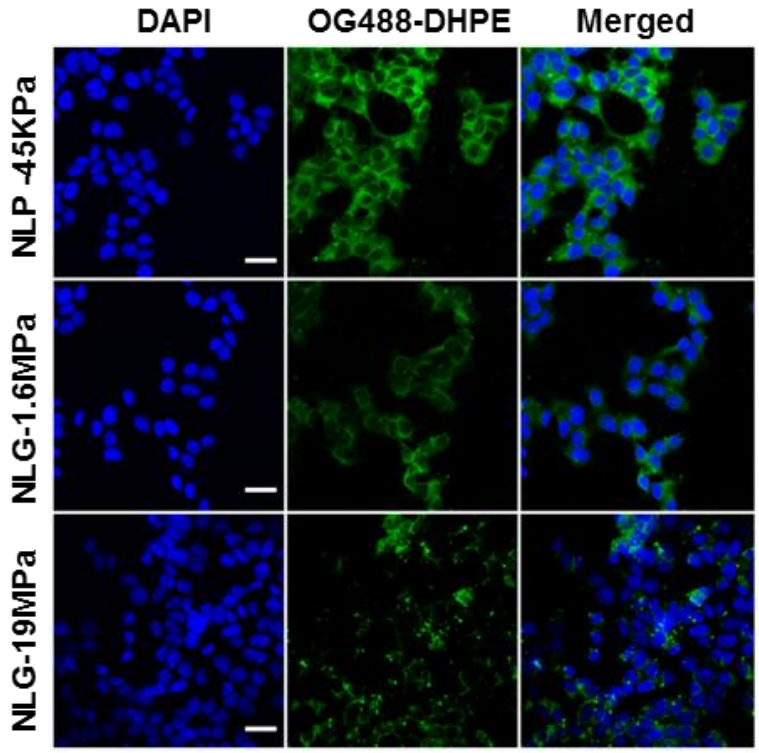

Supplementary Figure 3. Fluorescent microscope images of MCF7 cellular uptake of engineered NLP-45KPa, NLG-1.6MPa, and NLG-19MPa particles. The scale bars represent 50 μm.

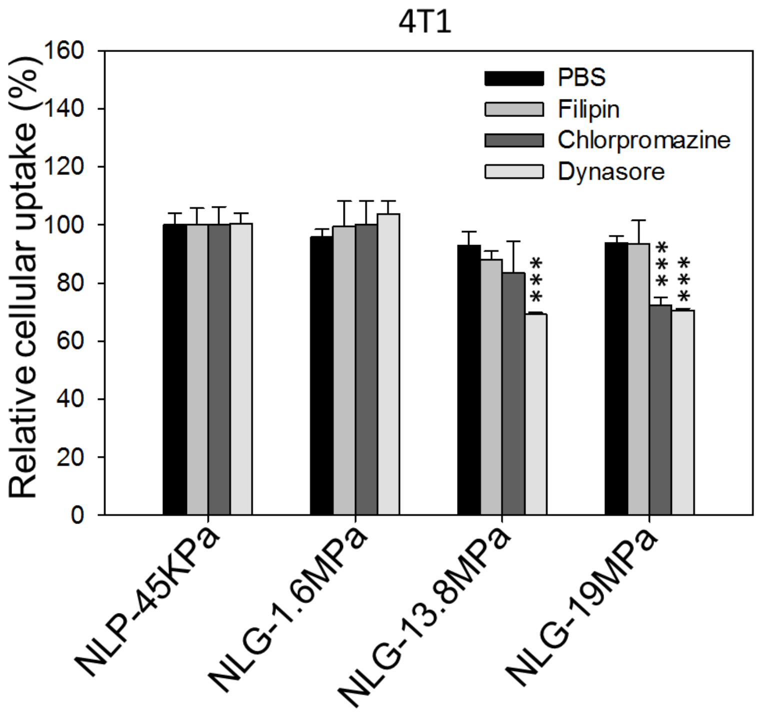

Supplementary Figure 4. The cellular uptake of NLP and NLGs with varying elasticity in murine neoplastic 4T1 cells in the presence of small molecules inhibiting clathrin (Chlorpromazine), caveolae (Filipin), and both endocytic pathways (Dynasore). *** $P<0.001$. The mean values and error bars are defined as mean and S.D., respectively

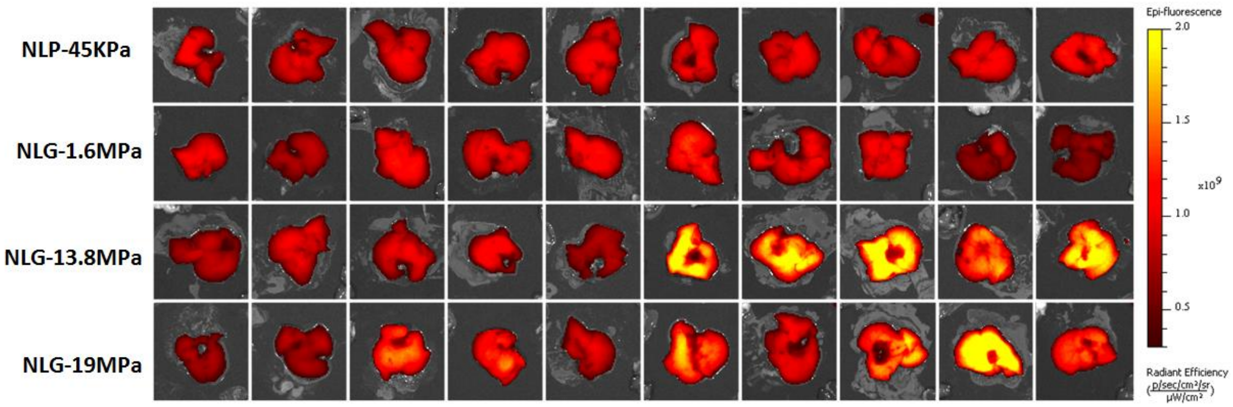

Supplementary Figure 5. *Ex vivo* NIR fluorescent images of excised livers at post-48 h (n=10 for each group).

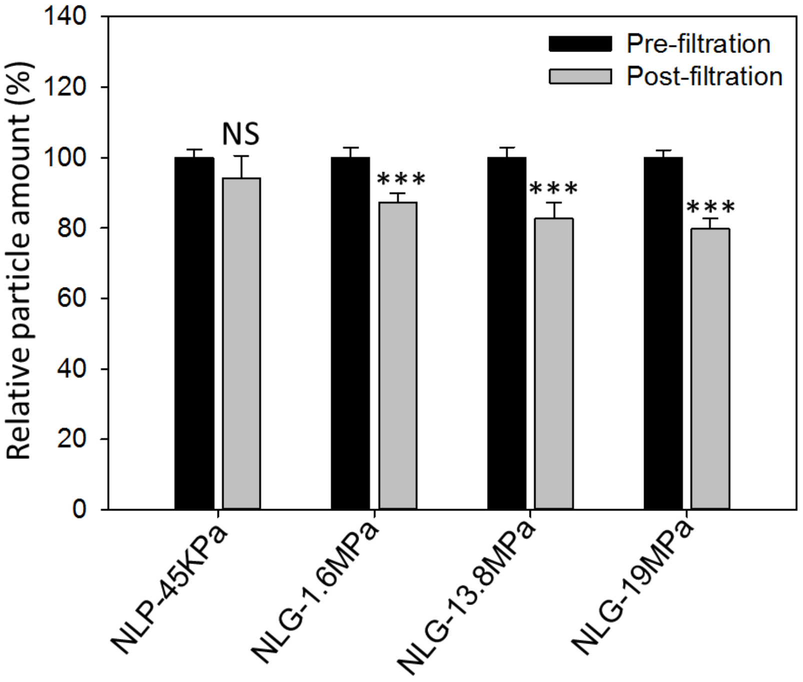

Supplementary Figure 6. The extravasation capability of NLP and NLGs with varying elasticity evaluated via the nanoporous membrane deformability assay. Four formulations of DiR-labeled NLP and NLGs (NLP-45KPa, NLG-1.6MPa, NLG-13.8MPa, and NLG-19MPa) were separately suspended in DMEM with 10% FBS at the concentration of 6.7×10^6 particles/mL. 1.5 mL of each DiR-labeled NLP and NLG solution was filtered through a 25mm Whatman Nuclepore Track-Etched Membranes with pore sizes of 100 nm using a syringe filtration holder. The NIR fluorescence intensity of nanoparticle solution of pre- and post-filtration was quantified using a Synergy H4 Microplate reader (Winooski, VT, USA) at excitation/emission wavelengths of 750 and 780 nm, respectively. The extravasation capability of NLP and NLGs with varying elasticity evaluated via the nanoporous membrane deformability assay. *** $P<0.001$. The mean values and error bars are defined as mean and S.D., respectively.

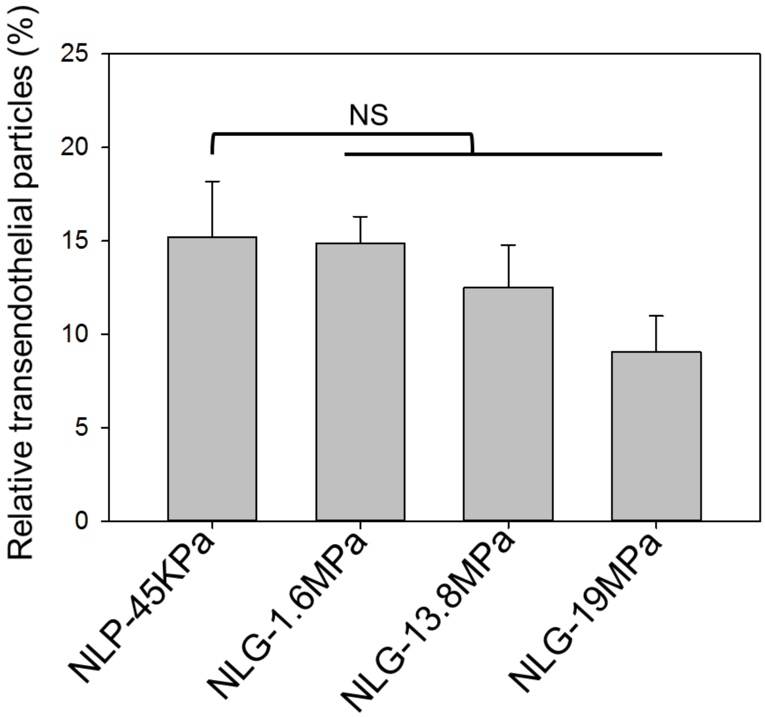

Supplementary Figure 7. NLP and NLGs translocation through the endothelial barrier was analyzed using endothelial barrier permeability assay. In order to quantify the vascular permeability of DiR-labeled NLP and NLGs (NLP-45KPa, NLG-1.6MPa, NLG-13.8MPa, and NLG-19MPa), we constructed an *in vitro* endothelial barrier by seeding 20,000 HMVECs on attachment factor-coated transwell insert with 3 μm pores and incubated the inserts for 48 hours. An HMVEC-coated transwell insert was then incubated in the conditioned media of human breast cancer MDA-MB-231 cells for 24 hours to induce vascular permeability. 100 μL of NLP or NLGs in DMEM (6.7×10^6 particles/mL) and 600 μL of DMEM were separately added to the top and bottom sides of the HMVEC-coated transwell inserts and further incubated for 4 hr. The DMEM in the bottom chamber was collected and DiR-labeled NLP or NLGs translocated through the endothelial barrier was quantified by measuring their fluorescence using a Synergy H4 Microplate reader at excitation/emission wavelengths of 750 and 780 nm, respectively. The transendothelial particle number was calculated based on the fluorescence. NS, not significant. The mean values and error bars are defined as mean and S.D., respectively.

Supplementary References

1. Liang, X., Mao, G. & Ng, K. Y. S. Mechanical properties and stability measurement of cholesterol-containing liposome on mica by atomic force microscopy. *J. Colloid Interface Sci.* **278,** 53–62 (2004).
2. del Pino, P. *et al.* Basic Physicochemical Properties of Polyethylene Glycol Coated Gold Nanoparticles that Determine Their Interaction with Cells. *Angew. Chem. Int. Ed.* **55,** 5483–5487 (2016).
3. Guo, P. *et al.* ICAM-1 as a molecular target for triple negative breast cancer. *Proc. Natl. Acad. Sci.* **111,** 14710–14715 (2014).
4. Yang, J. *et al.* Lipocalin 2 promotes breast cancer progression. *Proc. Natl. Acad. Sci.* **106,** 3913–3918 (2009).
